# Impact of Yeast Types on Quality Characteristics and Storage Stability of Industrial Kefir

**DOI:** 10.1002/fsn3.70238

**Published:** 2025-05-05

**Authors:** Oktay Yerlikaya, Asli Akpinar, Derya Saygili

**Affiliations:** ^1^ Faculty of Agriculture, Department of Dairy Technology Ege University Izmir Turkiye; ^2^ Engineering Faculty, Food Engineering Department Manisa Celal Bayar University Manisa Turkiye; ^3^ Izmir Kavram Vocational School Culinary Program Izmir Turkiye

**Keywords:** kefir, rheological properties, sensory analysis, shelf life, yeasts

## Abstract

Kefir is a fermented dairy product that contains many types of lactic acid bacteria and different types of yeast. Yeasts in kefir culture are important in creating the unique properties of kefir. When yeasts are not included in kefir production, the unique properties of kefir do not emerge. In this study, the effects of various yeast microorganisms that can cause negative effects, such as swelling and deformation, in industrial kefir production on product quality characteristics were investigated. For this purpose, a commercial yeast‐free kefir culture was supplemented with selected yeasts: *Kluyveromyces marxianus* (KM), *Debaryomyces hansenii* (DH), *Kluyveromyces lactis* (KL), *Saccharomyces boulardii* (SB), *Saccharomyces cerevisiae* (SC), *Candida colliculosa* (CC), *Yarrowia lipolytica* (YL), and *Geotrichum candidum* (GC) used in kefir production. Some physicochemical, rheological, microbiological, and sensory properties of the kefirs were examined during 28 days of storage. As a result of the research, the highest hardness and viscosity values (131.00 ± 1.41 g—1236.50 ± 47.38 cp) were observed in kefirs containing *G. candidum*, while the highest CO_2_ amount (151.40 ± 1.07 mg/100 mL) was determined in kefirs containing *S. cerevisiae* (*p* < 0.05). While serum separation was not determined in any sample, the highest water holding capacity (58.00% ± 00%) was determined in kefirs containing *Y. lipolytica*. In terms of sensory evaluation, kefir enriched with *C. colliculosa* received the highest overall acceptance. In general, the use of different yeast types in kefir production is of great importance, especially in terms of gas—carbon dioxide (CO_2_) formation and product quality.

## Graphical abstract

Kefir is a fermented dairy product that contains many types of lactic acid bacteria and different types of yeast. Due to the CO_2_ released as a result of the fermentation of yeast, deformities and swelling occur in the packaging of the products, which creates a problem because it is perceived as damaged by consumers. The use of different yeast species together with kefir culture in the production of kefir beverage affected the physicochemical, microbiological, some functional and sensory properties of the products.

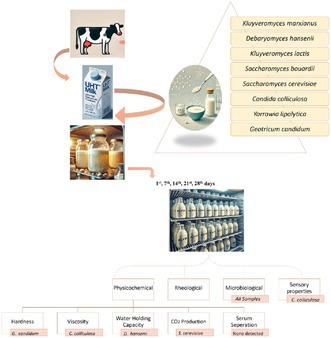

## 1 Introduction

There are a variety of commercially produced fermented dairy products around the world. Kefir, one of these fermented milk products, is produced and offered for sale by commercial companies. Kefir is widely acknowledged as a prominent product among probiotic beverages (Voidarou et al. 2020). According to Market research (2024), the Compound Annual Growth Rate (CAGR) of 6.36% is predicted for the Kefir Market, which was valued at USD 4.08 billion in 2023 and is projected to grow to USD 4.33 billion in 2024, reaching USD 6.28 billion by 2030 (Chandan and Kilara 2013; Devaraj and Baharuddin 2024). Fermented milk is a diverse group of products with distinct structures, appearances, and flavors, which are produced through the fermentation of milk by specific microorganisms, particularly lactic acid bacteria (Robinson et al. 2002). It is known that the lyophilized kefir cultures sold in the market for industrial kefir production and marketed by commercial companies contain very little or no yeast. The presence of yeast in kefir can lead to CO_2_ accumulation during fermentation, resulting in packaging swelling and potentially leading consumers to perceive the product as spoiled. Consequently, the incorporation of yeast is often restricted in commercially produced kefir. For these reasons, kefir cultures produced for commercial purposes contain some lactic acid bacteria, and these products do not have a foamy structure as in traditional kefir, and their ethyl alcohol content is also very low (Wszolek et al. 2006; Nielsen et al. 2014; Arslan 2015).

The fact that industrially produced kefir is more viscous and has less yeast taste than conventionally produced kefir is due to the differences in the microorganisms used in the fermentation of industrially and conventionally produced kefirs (Tomar et al. 2019). Studies show that the sensory properties of kefir produced using kefir grains are better than those of kefir produced using kefir starter culture (Leite et al. 2013; Tomar et al. 2019; Alves et al. 2021). However, kefir produced using a starter culture has lower titratable acidity and less serum separation. While the shelf life of kefir produced using kefir grains varies between 3 and 12 days, the shelf life of kefir produced using commercial kefir starter culture can be up to 28 days (Bengoa et al. 2019). Since kefir produced using traditional methods is more appreciated by consumers than kefir produced using industrial methods, studies on the use of modern techniques and methods to obtain products with properties similar to those of kefir produced by this method are increasing (Kim et al. 2018). This difference in taste is due to the different bacterial species and the presence of different yeast microorganisms in kefir produced with kefir grains (Farnworth and Mainville 2003; Singh and Shah 2017; Azizi et al. 2021).

Considering that both yeasts and lactic acid bacteria (LAB) in kefir grains significantly contribute to the taste, aroma, and texture of traditional kefir, the development of starter cultures for industrial production should incorporate yeast strains capable of controlled CO_2_ production (Cerff 2002; Farnworth and Mainville 2003; Kabak and Dobson 2011).

Yeasts have an important role in kefir fermentation as they produce ethanol and CO_2_ (Erten et al. 2014; Turkmen 2017; Maicas 2020). Kefir granules generally contain lactose‐fermenting yeasts (*Kluyveromyces lactis*, *Kluyveromyces marxianus*, and *Torula kefir*) as well as yeasts that cannot ferment lactose ( *Saccharomyces cerevisiae* ) (Angulo et al. 1993; Loretan et al. 2003). Yeasts are unicellular, spherical or oval‐shaped fungi that are ubiquitous in nature. They exhibit facultative anaerobic metabolism, enabling them to grow under both aerobic and anaerobic conditions by utilizing oxygen or fermentable organic substrates as electron acceptors. When they use O_2_, they convert carbohydrates into CO_2_ and H_2_O, while when they do not use O_2_, they create ethanol and CO_2_. Species such as *K. marxianus* and *Saccharomyces* spp. are the most common ones in fermentative spoilage (Varham and Sutherland 1994). However, in dairy products such as kefir and kumiss, yeasts contribute to the fermentation process. It has been reported that yeasts play an important role in the development of symbiosis between microorganisms in kefir grains (Ottagalli et al. 1973; Azizi et al. 2021).

There is limited research on the quality effect of using different yeasts in kefir production. The main desired feature of kefirs produced with kefir starter culture is to obtain the taste, aroma, and texture closest to traditional kefirs produced with kefir grains (Barukčić et al. 2017; Hong et al. 2019). In order to obtain the closest flavor and structure to traditional kefir produced using kefir grains, microorganisms used as starters and especially yeast species or strains found in the natural microbiota of kefir grains should be used. Kefir's unique cooling and relaxing effect is caused by yeast‐derived CO_2_ production (Bengoa et al. 2019; González‐Orozco et al. 2022). Although the presence of a certain level of CO_2_ is an important criterion for the formation of sensory properties specific to kefir, its production in large quantities can also cause negative quality characteristics, such as packaging deformation and excessive foaming (Farag et al. 2020; Yilmaz et al. 2022).

Since the development and metabolic activities of yeasts cannot be limited like bacteria during the storage of kefir, an increase in the amount of CO_2_ is observed because of yeast activity during subsequent storage. Although bacterial growth and metabolic activity are also not entirely inhibited during storage, their impact is less detrimental compared to yeasts, as CO_2_ production by bacteria is typically minimal (Lopitz‐Otsoa et al. 2006; Azizi et al. 2021). The main issue with CO_2_ is the swelling and even explosion of the containers. Considering that excessive CO_2_ production will cause some negative taste and aroma defects in kefir, it is thought that the increase in CO_2_ should be controlled. For this purpose, the amount of yeast in starter cultures obtained from kefir grains is limited, and kefir production can be done in a controlled manner through starter culture (Farag et al. 2020; Maicas 2020).

Studies on the effect of the use of different yeasts in kefir production on the quality of the beverage are limited (Ivanova et al. 2012; Agarbati et al. 2021). This study focused on the effects of different species and strains with the potential to be used industrially on the quality characteristics of dairy products. Yeast‐free commercial kefir culture was combined with different yeast species/strains, and some physicochemical, microbiological, and sensory properties of kefir produced with these cultures were investigated.

## 2 Materials and Methods

### 2.1 Materials

UHT milk used as a raw material in kefir production was purchased from Mis Süt A.Ş. Kefir culture, devoid of yeast microorganisms, was obtained from Referans Food Company (MicroMilk Srl.). Yeast species and strains were sourced from Ege University, Faculty of Agriculture, Dairy Technology Microbiology Laboratory, and various culture companies. The KFA/A1 culture used contains the following microorganisms: *Streptococcus thermophilus* , *Lactococcus lactis* subsp. *lactis*, *Lactococcus lactis* subsp. *cremoris*, *Lactococcus lactis* subsp. *lactis* biovar *diacetylactis*, *Leuconostoc mesenteroides* subsp. *cremoris*. Yeast species and strains are *Kluyveromyces marxianus* LAF 4 (coded as KM) (Chr. Hansen), *Debaryomyces hansenii* LAF 3 (coded as DH) (Chr. Hansen), *Kluyveromyces lactis* PYCC 4356 (coded as KL), *Saccharomyces boulardii* CNCM I‐745 (coded as SB), *S. cerevisiae* (coded as SC) (Yuvam yeast), *Candida colliculosa* LAF 7 (coded as CC) (Chr. Hansen), *Yarrowia lipolytica* NCAIM Y00591 (coded as YL), *Geotrichum candidum* GEO (coded as GC) (MicroMilk Srl.), and 200 mL packages (CoverisTM Rigid, Turkiye) were used for the storage of kefirs.

### 2.2 Methods

**FIGURE 1:**
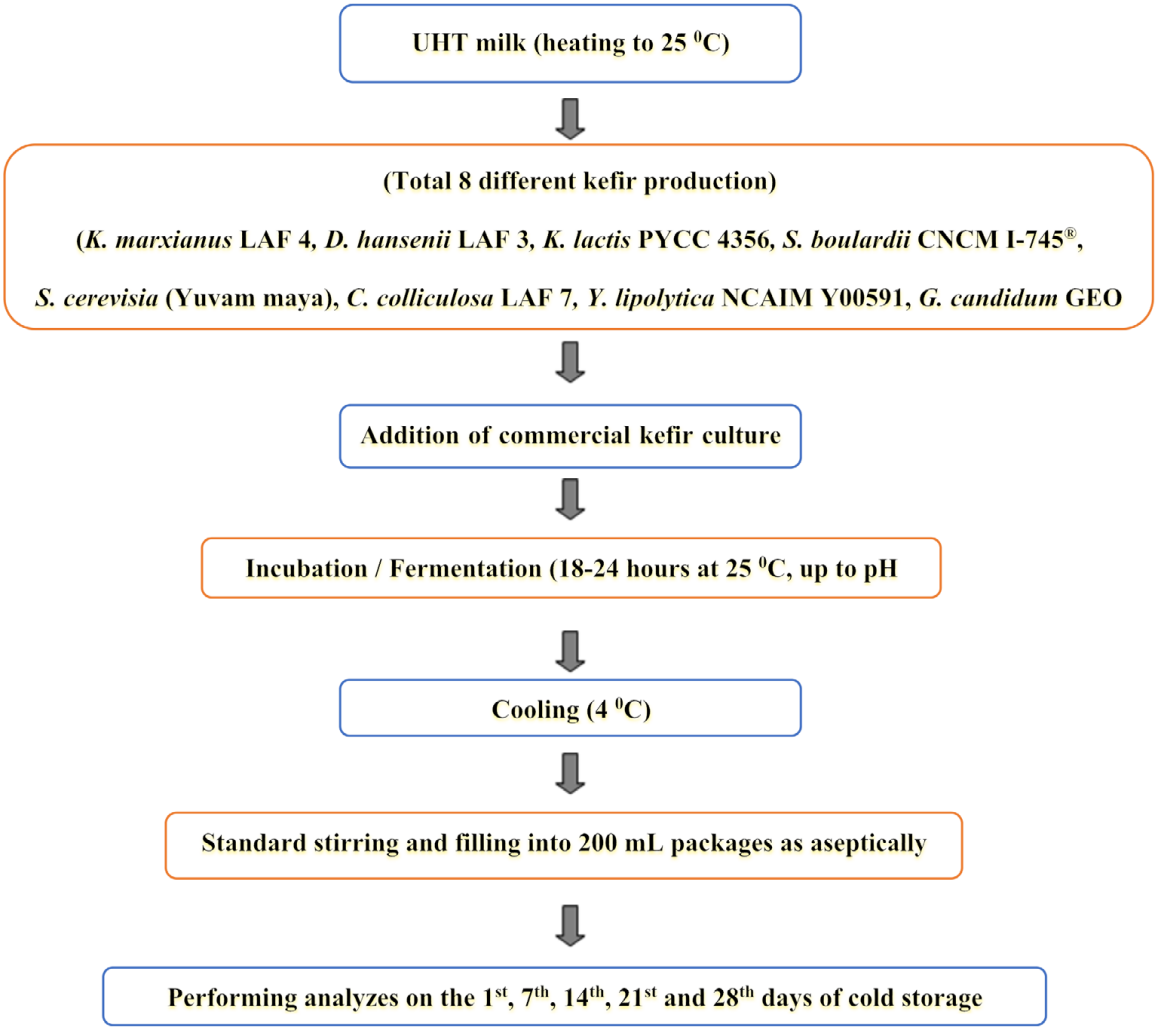
Kefir production flow diagram.

The production of kefir was carried out in Ege University, Dairy Technology Microbiology Laboratory of the Faculty of Agriculture (Figure 1). Yeasts were added concurrently with the kefir starter culture. Eight different yeast species, as specified in the materials section, were individually incorporated into the kefir culture, resulting in the production of eight distinct kefir samples. The kefir starter culture was weighed in the recommended amounts in accordance with the manufacturer's instructions (0.05 g/L) and activated for 4 h. Commercial yeasts obtained from Chr. Hansen and Micromilk Srl. were added following the manufacturer's instructions (0.01 g/L), and other yeasts were activated in Tryptic Soy Broth (Fluka) for 48–72 h, and their optical density was adjusted to 0.5 MacFarland turbidity at OD_600_. It was ensured that the kefir yeast amount was at least 10^3^ CFU/mL at the beginning of storage. For this, yeast cultures were taken to standard turbidity and were inoculated at a rate of 0.5% together with the starter culture into UHT milk to be processed into kefir. It was not necessary to produce a yeast‐free control group since a lactic milk beverage without yeast would not meet the definition of kefir. Analyses such as dry matter, fat, and protein were determined only on the 1st day after fermentation, while other analyses were determined on the 1st, 7th, 14th, 21st, and 28th days of storage.

### 2.3 Physicochemical Analyses Performed on UHT Milks

Dry matter, fat, protein, acidity, pH, and ash analyses were performed on the UHT milk used in kefir production. Dry matter values were obtained by gravimetric method in Binder/Germany brand oven AOAC (1997); fat values were analyzed by Gerber method with Funke Gerber/Germany brand Gerber centrifuge according to ISO 2446‐IDF 226 (2008); Protein values were determined by Kjeldahl method with Velp Scientifica DK 20 brand protein determination device IDF (1993) and Gerhardt/Germany brand distillation with AOAC (1997); acidity (% lactic acid) values were determined by TSE 1330 (Anonymous 1999); pH values were determined with a Hanna Instruments 211 brand digital pH meter. The ash value was obtained by incinerating the samples at 550°C (AOAC 2000). The total carbohydrate was calculated by a different method using this formula: [total solids − (protein + fat + ash)] (Wszolek et al. 2001).

### 2.4 Physicochemical Analyses Performed on Kefir Drinks

#### 2.4.1 Determination of Dry Matter

To determine the dry matter values of the kefir samples, 5 g of homogenized kefir was weighed by mixing it in the dry matter container, dried in a drying cabinet at 100°C–105°C, cooled in a desiccator, and weighed again. The % dry matter amount was calculated by taking the difference with tare (Kurt et al. 1993).

#### 2.4.2 Fat Analysis

Fat values in kefir were determined by the Gerber method. For each sample, 10 mL of sulfuric acid with a density of 1.820 was placed in the butyrometer, then 11 mL of kefir sample was added, and 1 mL of amyl alcohol was added and centrifuged for 5 min at 1100 rpm in the centrifuge. After the butyrometers were kept in a water bath at 65°C for 5 min, the amount of fat was determined as a percentage.

#### 2.4.3 Protein Determination

Total nitrogen was determined by the Kjeldahl method. One gram of the sample was taken and placed in the Kjeldahl flask, a catalyst tablet was added, and the combustion temperature was gradually increased to 380°C–400°C. The burning process continued until the sample solution turned clear green, and after the clear color appeared, the burning process continued for another 30 min. After the sample came to room temperature, 50 mL of pure water was added and placed in the distillation section, and the distillate was collected in 25 mL of 4% boric acid solution. 0.1 N HCl was used in the titration. Milk protein (%) was calculated by multiplying the determined nitrogen amount by 6.38.

#### 2.4.4 Ash Determination

Ash values were determined by weighing approximately 5 g samples in porcelain crucibles, burning at 550°C, cooling in a desiccator, and then weighing (Association of Official Analytical Chemists (AOAC 2000)).

#### 2.4.5 Total Carbohydrate Analysis

The total carbohydrate (lactose) content was determined by the lactose calculation method (Wszolek et al. 2001).

#### 2.4.6 pH and Titratable Acidity Analysis

pH measurement was detected after calibration using a HANNA Instruments pH 211 Microprocessor brand pH meter and a probe designed for fermented dairy products. To determine titratable acidity, after adding 0.5 mL of 1% phenolphthalein to 25 mL kefir samples, titration was performed with 0.1 N NaOH until a permanent light pink color was formed (pH 8.3; Bradley et al. 1992).

#### 2.4.7 Viscosity

Although evaluation is made with multiple speed parameters in Newtonian type flow beverages, the viscosity values of kefir samples were determined using a Brookfield viscometer (Model DV II + Pro, Brookfield Engineering Laboratories Inc., Middleboro, MA, USA) with different speed and spindle selections (Tork: 10%–90%) for the most suitable conditions for all samples. The most suitable spindle (LV‐2) and speed (120 rpm) parameters were selected for the analysis, and the analysis was carried out with the sample temperature being 4°C. The results were given as centipoise (cP) (Yerlikaya et al. 2021).

#### 2.4.8 Hardness

The hardness values of kefir samples were determined by using the appropriate probe (TA4/1000 probe, 38.1 mm diameter cylinder acrylic) and printing speed using Brookfield CT3 Texture Analyzer. The conditions adopted in the analysis are as follows: target value: 4500 g, trigger load: 6.8 g, test speed: 0.50 mm/s, and probe penetration: 1 cm. Following the graphs obtained, calculations were made using the Brookfield Texture Pro CT V 1.2 software program. The hardness values are given in grams as the maximum force applied to the sample (Yerlikaya et al. 2020).

#### 2.4.9 Serum Separation

Kefir samples were placed in 100 mL graduated cylinders and stored for 28 days at 4°C. Serum separation was given as % serum separation by reading from the top of the cylinder downward on the 1st, 7th, 14th, 21st, and 28th days of storage (Paraskevopoulou et al. 2003).

#### 2.4.10 Water Holding Capacity

Water holding capacity (WHC) was determined by modifying the method reported by Remeuf et al. (2003). For this, 25 g of kefir samples were weighed into a centrifuge tube, centrifuged at 6000 g at 10°C for 10 min, and then the serum remaining on top was poured. The remaining portion was weighed (final weighing), and the water retention capacity value was calculated using the equation below.

$$\begin{array}{c} \mathrm{WHC}\;\left(\%\right)=[\left(\text{final weighing}-\text{weight of centrifuge tube}\right)\\ /\text{sample amount}]\times 100 \end{array}$$

#### 2.4.11 Determination of CO_2_

The method is based on the principle that some of the NaOH solution added to the sample binds with CO_2_ and turns into Na_2_CO_3_, and the remaining amount of NaOH is determined (Anonymous 1976; American Public Health Association (APHA) 1978). Unopened bottles and glassware were kept at 0°C to prevent CO_2_ release in the samples. Thirty milliliters of 0.1 N NaOH solution and 3 mL of BaCl_2_ solution were placed in a conical flask, and 10 mL of sample was carefully pipetted and slowly discharged so that its tip touched the bottom of the solutions in the conical flask. Two drops of thymolphthalein indicator were added to the solution, shaken well, and titrated with 0.1 N HCl acid solution until the blue color disappeared. The same process was performed by adding a 10 mL kefir sample, the CO_2_ of which had been boiled (blind experiment).

##### 2.4.11.1 Calculations

$$1\;\mathrm{mL}\;0.1\;N\;\mathrm{HCl}=0.0022\;g\;{\mathrm{CO}}_{2}$$

$$\text{Amount of}\;{\mathrm{CO}}_{2}\;\mathrm{mg}/100\;\mathrm{mL}=\left[\left(A-B\right)/m\right]\times 0.0022\times 100\times 1000$$

A = Volume of 0.1 N NaOH bound by CO_2_ = 30‐C; B = Blind (example of kefir with CO_2_ removed by boiling).

#### 2.4.12 Acidity Index

In order to control the oxidation that may result from the use of different yeast species in kefir samples, 25 mL of ether‐ethanol (2:1) mixture was added to 1 mL of kefir. Titration was performed with 0.1 N KOH by dropping 2% phenolphthalein, and the consumption was recorded as KOH/g (Oliveira et al. 2018).

$$\mathrm{FFA}\;\left(\%\right)=\left(\mathrm{mL}\;\text{titration}\times \text{Molarity of}\;\mathrm{KOH}\times 28.2\right)/g$$

#### 2.4.13 Microbiological Analyzes

Ten milliliter of kefir sample was homogenized and mixed with 90 mL sterile (0.1% peptone water) dilution liquid, and microorganisms were counted using the following methods:

##### 2.4.13.1 Count of *S. thermophilus/Lactococcus* spp

M17‐lactose Agar was used to enumerate lactococci and *S. thermophilus* . The seeded Petri dishes were incubated under aerobic conditions at 37°C for 48 h (Terzaghi and Sandine 1975).

##### 2.4.13.2 Count of *Leuconostoc mesenteroides* subsp. *cremoris*

The starter culture used in the production of kefir does not contain any *Lactobacillus* species to count *L. cremoris*; it was inoculated on MRS agar (Mathot et al. 1994) containing 30 μg/mL vancomycin (Sigma V‐2002) using the cultural counting technique, and the Petri dishes were incubated anaerobically for 72 h at 30°C. At the end of incubation, white and opaque colonies were evaluated.

##### 2.4.13.3 Total Yeast and Mold Count

To determine the total yeast and mold counts, Yeast Extract Chloramphenicol Agar (Merck, Germany) was inoculated in the medium and the plates were incubated at 25°C under aerobic conditions for 3–5 days. Colonies formed in the medium at the end of the incubation period were evaluated as yeast and mold according to their reproductive characteristics (Witthuhn et al. 2005).

##### 2.4.13.4 Sensory Evaluation

A 9‐point hedonic scale was used for the sensory evaluation of the kefir samples. A panel of eight trained individuals (four women and four men) with experience in sensory analysis assessed the samples based on consistency, odor, taste, aroma, and overall acceptability. The kefir samples were presented in their original 200 mL packages during the evaluation (Bodyfelt et al. 1998; Zhi et al. 2016).

##### 2.4.13.5 Statistical Evaluation

The study was performed in three replicates, and each analysis was conducted in two parallels. One‐way analysis of variance (One‐way ANOVA) was performed to determine the differences between the properties of the produced kefirs and the effects of storage time on the samples. For this purpose, the SPSS version 20.00 (SPSS Inc. Chicago, Illinois) statistical analysis program was used, and the data whose statistical difference were found to be significant as a result of the analysis of variance were analyzed at the *p* < 0.05 significance level in the Duncan multiple comparison test.

## 3 Results and Discussion

### 3.1 Properties of UHT Milk

The physicochemical properties of UHT milk obtained from a commercial company are shown in Table 1. The product properties of the milk used in the production of kefir have a significant impact on the physicochemical properties of the products. It is important to use milk with a standard composition in each product in order to ensure that the developed products have a standard composition and to reveal the effect of the difference in yeast used in the products after fermentation. The effects of different yeast microorganisms and culture bacteria were better demonstrated by using standardized and homogenized UHT milk. Since production was carried out under aseptic conditions, contamination was not allowed. In this respect, the effects of different yeast species and strains were better demonstrated (Metin 1998; Oner et al. 2010; Dag et al. 2021).

**TABLE 1:** Some properties of UHT milk used in the production of kefir drinks (*n* = 3).

	pH	Acidity (LA%)	Dry matter (%)	Lactose–total carbohydrate (%)	Fat (%)	Protein (%)	Ash (%)
UHT milk	6.70 ± 0.00	0.135 ± 0.00	12.01 ± 0.02	5.68 ± 0.03	2.80 ± 0.00	2.88 ± 0.04	0.65 ± 0.01

### 3.2 Physicochemical Properties

The physicochemical properties of kefir produced using different yeasts are given in Table 2. Statistical analysis indicated that the differences among the samples were significant in terms of dry matter, protein, and total carbohydrate content (*p* < 0.05), while no significant differences were observed in fat and ash content (*p* > 0.05).

**TABLE 2:** Dry matter, protein, fat, total carbohydrate, and ash contents of kefir drinks after fermentation (%) (*n* = 3).

	Kefir samples							
	DH	KM	CC	YL	SB	SC	KL	GC
Dry matter (%)	11.19 ± 0.00^YZ^	11.23 ± 0.02^YZ^	11.14 ± 0.01^XY^	11.23 ± 0.03^YZ^	11.14 ± 0.02^XY^	11.18 ± 0.02^XYZ^	11.24 ± 0.03^Z^	11.09 ± 0.09^X^
Protein (%)	2.75 ± 0.01^XY^	2.88 ± 0.01^Z^	2.70 ± 0.01^X^	2.80 ± 0.04^Y^	2.73 ± 0.06^XY^	2.94 ± 0.03^Z^	2.74 ± 0.05^XY^	2.73 ± 0.01^XY^
Fat (%)	2.70 ± 0.00	2.75 ± 0.07	2.65 ± 0.07	2.60 ± 0.00	2.65 ± 0.07	2.75 ± 0.07	2.60 ± 0.00	2.60 ± 0.00
Total carbohydrate lactose (%)	5.16 ± 0.02^YZ^	4.91 ± 0.06^X^	5.12 ± 0.02^Y^	5.17 ± 0.04^YZ^	5.13 ± 0.10^YZ^	4.82 ± 0.09^X^	5.27 ± 0.04^Z^	5.15 ± 0.05^YZ^
Ash (%)	0.59 ± 0.01	0.69 ± 0.01	0.67 ± 0.03	0.66 ± 0.03	0.63 ± 0.01	0.67 ± 0.03	0.64 ± 0.04	0.62 ± 0.05

*Note:* ^X,Y,Z,T,K,L,M,N^: Different letters on the same line indicate difference at *p* < 0.05 level.

Abbreviations: CC, *Candida colliculosa* LAF 7; DH, *Debaryomyces hansenii* LAF 3; GC, *Geotrichum candidum* GEO; KL, *Kluyveromyces lactis* PYCC 4356; KM, *Kluyveromyces marxianus* LAF 4; SB, *Saccharomyces boulardii* CNCM I‐745; SC, *Saccharomyces cerevisiae*; YL, *Yarrowia lipolytica* NCAIM Y00591.

In addition to the structure, sensory, and microbiological properties of kefir, the dry matter content of the product is of great importance on its textural properties. The dry matter content of kefir varies between 11.24% and 11.09%. The lowest dry matter content is seen in the GC sample, while the highest is in the KL sample. It was determined that there were similarities between other samples, but some samples also showed similarities with products (KL and GC) with dry matter contents close to the upper value (DH, KM, YL, and SC) and near the lower value (SC, CC, and SB). The dry matter values obtained in the study were different from the studies of Yildiz‐Akgul et al. (2018), Kezer (2013), Cinar (2019), and Dag et al. (2021) working on kefir, and were found to be similar to the values obtained from the studies of Irigoyen et al. (2005), Ufakseker (2019), Barazi (2022), and Eryilmaz et al. (2022). These differences are due to the different standardization, raw material, and enrichment applications of the researchers in kefir production. The change in dry matter between products varies depending on the composition and breakdown of sugar into lactic acid and other metabolites as a result of fermentation, depending on the use of different starter cultures (Ching‐Yun and Ching‐Wen 1999). It is thought that the use of different yeast species causes differences in the dry matter content of the products due to the metabolization of different carbohydrate sources and the production of the resulting metabolites (Pouris et al. 2024).

The SC sample exhibited the highest protein content, while the CC sample showed the lowest. The DH, SB, KL, and GC samples displayed comparable protein levels, falling within the lower to mid‐range. The KM sample had a slightly lower protein content than SC but remained relatively close in value. Although the protein content of the products varies between 2.94% and 2.70%, the protein content of the milk used in production does not vary much from the protein content of the products and is similar. Although standard raw materials are used in the production of all beverages, the metabolism characteristics of different yeast species and strains and their symbiotic relationships with kefir starters can cause protein degradation. In addition, the use of peptides formed due to protein degradation can also be slightly effective in protein content. When we look at the studies in the literature, Tomar and Akarca (2018) observed protein results close to the milk content used in their study and stated that the change in protein content seen in kefir depends on the milk composition used. Although the variation between the products in terms of fat content is seen as insignificant, it is observed that SC and KM samples have the highest fat content, and KL and YL samples have the lowest. The fat content of kefir varies between 2.75% and 2.60%. In his study, Barazi (2022) stated that the fat amount of kefirs produced varied between 2.82% and 2.62%, Dag et al. (2021) determined the fat amount of kefirs to be 2.88%–2.69%, Gursoy et al. (2020) reported that the ranges of dry matter, protein, and fat contents of commercial kefir samples were 9.49%–11.97%, 2.30%–3.44%, and 2.50%–3.00%, respectively, and Uslu (2010) reported the fat content of kefirs sold in Ankara–Turkey as 2.59%. Kalkan (2019) found the fat content of control and *S. boulardii‐*added kefir beverages to be 2.77% and 2.87%, respectively. The results of this study are similar to our study in terms of fat content. Although the production parameters are different, the results of this study are similar to our study in terms of fat content. It is observed that the fat content of kefir drinks is close to the fat content of the milk used in production.

Kefir undergoes both lactic acid and alcoholic fermentation through the metabolic activities of lactic acid bacteria and yeasts. These processes result in the production of various metabolites, including lactic acid, ethanol, carbon dioxide (CO_2_), and a range of aroma compounds (Rattray and O'Connell 2011). The lactose content in kefir is less than that in milk. Garcia‐Fontan et al. (2006) stated that the lactose content of kefir decreased from 4.92% to 4.02% in the first 24 h of fermentation. During fermentation, lactic acid bacteria and yeasts that can use lactose breakdown to form lactic acid and various metabolites. Some yeast species and strains that use the breakdown products of lactose use galactose and lactic acid to provide the product with its unique properties during the fermentation process. Although the total carbohydrate content of kefir varies between products, the KL sample has the highest content, and the SC sample has the lowest content. It can be seen that the KL sample has the highest value and the SC sample has the lowest value in terms of total carbohydrate content. While the GC, SB, YL, and DH samples are seen to be close to the upper value, the KM sample is similar to the lower value. The total carbohydrate content of kefir varies between 5.27% and 4.82%. Considering that the total carbohydrate amount of milk used in production is around 5.7%, it seems a rather high value for lactose in milk during fermentation, as in other studies in the literature. In this case, it is expected that the total carbohydrate amount of milk will decrease in kefir because of the breakdown that occurs during fermentation (Karagozlu and Kavas 2000; Irigoyen et al. 2005; Garcia‐Fontan et al. 2006; Hikmetoglu et al. 2020).

Although the change in the ash content of kefirs between products was determined to be not significant (*p* > 0.05), it was observed that the KM sample had the highest ash content, and the DH sample had the lowest ash content. The ash content of kefir varies between 0.69% and 0.59%. The values obtained in our study are similar to the ash amount of milk used and the literature (Karagozlu 1990; Kok‐Tas et al. 2014; Ufakseker 2019; Dag et al. 2021).

### 3.3 pH and Titratable Acidity

The pH and titratable acidity (TA) values of kefir samples are given in Table 3. When evaluated statistically, the change in pH values in all products throughout storage (*p* < 0.05) and among the products on each storage day was found to be significant (*p* < 0.05). As expected, the acidity of each kefir sample increased progressively during storage. By the 28th day, a rise of 0.08–0.12 units in pH was observed in the DH, KM, CC, YL, SB, and GC samples. This increase is likely attributed to the metabolic activity of yeasts, particularly the production and assimilation of organic acids such as lactic acid. While the highest pH values were seen in all samples on the first day of storage (pH 4.4), the lowest pH values were seen on the 21st day of storage. The decrease in pH values of kefir samples during storage, as expected, shows that each yeast works in harmony with lactic acid bacteria, and there is no negative interaction. Although the change occurring on each storage day varies between kefir samples, the reason for this change is due to the difference in the starter culture used in kefir production (Dag et al. 2021). Different yeast species used in the study caused different pH values of kefir. This situation may vary depending on whether different yeast species metabolize lactose and the ability to metabolize other organic acids formed. In addition, some yeasts can cause pH differences by metabolizing lactic acid. This situation is common in kefir beverages. On the first day of storage, the highest pH values were 4.47, and the lowest pH values were 4.37. While DH, CC, YL, SC, and GC samples show values close to each other, KM, KL, and SB samples have values close to each other. It was determined that the pH values of the kefir beverage containing *K. marxianus* (KM) were lower on the 7th day of storage, but the pH values of all samples were similar on the 14th day of storage. This is an indication that the fermentation process continues by yeasts. On the 21st day of storage, the decrease in pH values of all kefir samples continued, and the lowest pH values were observed in samples containing *K. marxianus*, *Y. lipolytica*, and *S. boulardii*.

**TABLE 3:** pH and acidity changes of kefir drinks during storage.

Storage days	Kefir samples							
	DH	KM	CC	YL	SB	SC	KL	GC
	pH							
1	4.46 ± 0.00^CZ^	4.37 ± 0.00^CX^	4.46 ± 0.00^CZ^	4.46 ± 0.01^DZ^	4.44 ± 0.01^EX^	4.47 ± 0.01^CZ^	4.44 ± 0.01^CY^	4.46 ± 0.00^EZ^
7	4.42 ± 0.00^BYZ^	3.35 ± 0.00^BCX^	4.44 ± 0.00^CZ^	4.42 ± 0.02^CYZ^	4.42 ± 0.00^DYZ^	4.41 ± 0.01^BY^	4.42 ± 0.02^CYZ^	4.44 ± 0.01^DZ^
14	4.33 ± 0.00^AXY^	4.33 ± 0.00^ABXY^	4.36 ± 0.00^BYZ^	4.35 ± 0.00^BZ^	4.34 ± 0.00^BXYZ^	4.35 ± 0.00^AZ^	4.32 ± 0.00^AX^	4.35 ± 0.00^BXYZ^
21	4.30 ± 0.00^AZ^	4.30 ± 0.01^AZ^	4.26 ± 0.00^AXY^	4.28 ± 0.00^AYZ^	4.24 ± 0.00^AXT^	4.34 ± 0.00^A^	4.34 ± 0.00^ABT^	4.31 ± 0.01^AZT^
28	4.41 ± 0.00^BY^	4.38 ± 0.01^BCXY^	4.38 ± 0.00^BXY^	4.36 ± 0.00^BX^	4.40 ± 0.00^CY^	4.35 ± 0.00^AX^	4.36 ± 0.00^BX^	4.40 ± 0.00^CY^
	Lactic acid (LA)%							
1	0.71 ± 0.00^A^	0.70 ± 0.00^A^	0.64 ± 0.00^A^	0.69 ± 0.00^A^	0.66 ± 0.00^A^	0.69 ± 0.00^A^	0.65 ± 0.00^A^	0.71 ± 0.00^A^
7	0.87 ± 0.04^DZ^	0.81 ± 0.01^BXY^	0.80 ± 0.01^BX^	0.80 ± 0.01^BX^	0.80 ± 0.00^BX^	0.81 ± 0.01^BXY^	0.82 ± 0.01^BXY^	0.85 ± 0.00^DYZ^
14	0.82 ± 0.00^BCZT^	0.82 ± 0.00^BZT^	0.79 ± 0.00^BX^	0.82 ± 0.00^CZ^	0.80 ± 0.00^BXY^	0.81 ± 0.01^BYZ^	0.79 ± 0.00^BX^	0.83 ± 0.01^CT^
21	0.84 ± 0.01^CDX^	0.95 ± 0.01^CK^	0.88 ± 0.02^DYZ^	0.85 ± 0.01^DXY^	0.86 ± 0.01^DXY^	0.89 ± 0.02^CZ^	0.91 ± 0.00^CZT^	0.93 ± 0.01^ETK^
28	0.78 ± 0.00^BX^	0.85 ± 0.04^BY^	0.84 ± 0.00^CY^	0.82 ± 0.00^CXY^	0.83 ± 0.01^CY^	0.81 ± 0.02^BXY^	0.91 ± 0.02^CT^	0.80 ± 0.01^BXY^

*Note:* ^A,B,C,D,E^: Different letters in the same column indicate differences at *p* < 0.05 level. ^X,Y,Z,T,K,L,M,N^: Different letters on the same line indicate difference at *p* < 0.05 level.

Abbreviations: CC, *Candida colliculosa* LAF 7; DH, *Debaryomyces hansenii* LAF 3; GC, *Geotrichum candidum* GEO; KL, *Kluyveromyces lactis* PYCC 4356; KM, *Kluyveromyces marxianus* LAF 4; SB, *Saccharomyces boulardii* CNCM I‐745; SC, *Saccharomyces cerevisiae*; YL, *Yarrowia lipolytica* NCAIM Y00591.

Dag et al. (2021) found that the pH values of kefir produced using grain and lyophilized starter culture were in the range of 4.33–4.77, and Oksuztepe et al. (2020) found that the pH value of 25 plain kefir samples varied between 4.2 and 4.6, and Kalkan (2019) found that the pH values of kefir samples with and without *S. boulardii* added were between 4.18–4.21 and 4.27–4.36, respectively.

### 3.4 Hardness, Viscosity, Serum Separation, and Water Holding Capacity

Hardness, viscosity, serum separation, and WHC values of kefir samples are given in Table 4. When the hardness values are examined, it is seen that the hardness value increases throughout storage in all kefir samples. This increase observed for all kefir samples containing different yeast species was found to be statistically significant (*p* < 0.05). However, while the difference between the hardness values of the samples on the 1st and 7th days of storage was insignificant (*p* > 0.05), the difference between the changes occurring on the ongoing storage days and the hardness values was reported to be significant (*p* < 0.05). While hardness values vary between 22.0 and 131.00 g for all kefir samples, the values obtained on the 21st and 28th days of storage for *Geo. candidum* are remarkable. It was observed that with the increasing hardness value, the general approval score obtained for the GC sample on the 21st and 28th days of storage decreased significantly.

**TABLE 4:** Hardness, viscosity, serum separation, and water holding capacity properties of kefir drinks.

Storage days	Kefir samples							
	DH	KM	CC	YL	SB	SC	KL	GC
	Hardness (g)							
1	22.00 ± 0.71^A^	23.50 ± 1.41^A^	22.25 ± 1.06^A^	22.25 ± 0.35^A^	22.25 ± 1.77^A^	22.50 ± 0.00^A^	22.75 ± 1.77^A^	22.50 ± 0.71^A^
7	40.50 ± 0.00^B^	42.50 ± 2.12^B^	43.25 ± 4.60^B^	40.50 ± 2.12^B^	43.50 ± 3.54^B^	43.00 ± 4.24^B^	40.75 ± 1.06^B^	43.00 ± 2.83^C^
14	45.00 ± 2.12^CZT^	50.00 ± 0.00^CL^	43.00 ± 0.00^BYZ^	43.25 ± 0.35^BCYZ^	47.50 ± 0.71^BCK^	46.00 ± 1.41^BCTK^	41.25 ± 1.06^BY^	38.75 ± 0.35^BX^
21	46.00 ± 1.41^CX^	56.00 ± 3.54^DY^	50.50 ± 0.00^CXY^	47.00 ± 2.83^CX^	48.75 ± 1.77^BCX^	48.00 ± 2.83^BCX^	47.00 ± 3.54^CX^	82.00 ± 0.00^DZ^
28	44.00 ± 1.41^CX^	77.75 ± 0.35^EZ^	51.50 ± 1.41^CY^	44.50 ± 0.71^BCX^	52.00 ± 1.41^CY^	52.25 ± 1.06^CY^	51.25 ± 0.35^CY^	131.00 ± 1.41^ET^
	Viscosity (cP)							
1	2596.50 ± 36.06^CY^	569.50 ± 21.92^AX^	670.50 ± 13.44^AX^	590.50 ± 41.72^AX^	614.50 ± 13.44^AX^	598.00 ± 53.74^AX^	683.50 ± 133.64^AX^	646.50 ± 23.33^AX^
7	3218.50 ± 9.19^DZ^	2680.50 ± 115.26^CY^	1997.50 ± 96.87^CX^	2104.00 ± 244.66^CX^	2393.00 ± 113.14^CXY^	2155.50 ± 212.84^CX^	2209.50 ± 129.40^CX^	2142.00 ± 376.18^CDX^
14	2399.50 ± 129.40^CXY^	2736.00 ± 138.59^CYZ^	2856.00 ± 151.32^DZ^	2456.00 ± 229.10^CXY^	2827.50 ± 2.12^DZ^	2406.50 ± 164.76^CXY^	2550.00 ± 130.11^DXYZ^	2374.00 ± 24.04^DX^
21	1065.50 ± 154.86^BXY^	1443.00 ± 65.05^BYZ^	1090.00 ± 42.43^BXY^	1114.00 ± 35.36^BXY^	1078.00 ± 104.65^BXY^	1115.50 ± 321.73^BXY^	1002.50 ± 33.23^BX^	1602.50 ± 415.07^BCZ^
28	478.00 ± 106.07^AX^	1217.50 ± 157.68^BY^	530.00 ± 36.77^AX^	489.50 ± 21.92^AX^	531.50 ± 9.19^AX^	588.00 ± 26.96^AX^	627.50 ± 19.09^AX^	1236.50 ± 47.38^ABY^
	Serum separation (mL)							
1	0.00 ± 0.00	0.00 ± 0.00	0.00 ± 0.00	0.00 ± 0.00	0.00 ± 0.00	0.00 ± 0.00	0.00 ± 0.00	0.00 ± 0.00
7	0.00 ± 0.00	0.00 ± 0.00	0.00 ± 0.00	0.00 ± 0.00	0.00 ± 0.00	0.00 ± 0.00	0.00 ± 0.00	0.00 ± 0.00
14	0.00 ± 0.00	0.00 ± 0.00	0.00 ± 0.00	0.00 ± 0.00	0.00 ± 0.00	0.00 ± 0.00	0.00 ± 0.00	0.00 ± 0.00
21	0.00 ± 0.00	0.00 ± 0.00	0.00 ± 0.00	0.00 ± 0.00	0.00 ± 0.00	0.00 ± 0.00	0.00 ± 0.00	0.00 ± 0.00
28	0.00 ± 0.00	0.00 ± 0.00	0.00 ± 0.00	0.00 ± 0.00	0.00 ± 0.00	0.00 ± 0.00	0.00 ± 0.00	0.00 ± 0.00
	Water holding capacity (WPC) (%)							
1	62.80 ± 0.57^D^	61.00 ± 0.28^D^	62.20 ± 1.41^C^	62.00 ± 0.57^D^	60.80 ± 0.00^E^	59.60 ± 0.00^C^	61.00 ± 1.98^C^	60.80 ± 0.00^D^
7	52.40 ± 0.28^AY^	53.40 ± 0.00^AZ^	53.80 ± 0.00^AZ^	55.60 ± 0.00^AT^	52.80 ± 0.00^AY^	53.90 ± 0.42^AZ^	51.30 ± 0.42^AX^	52.50 ± 0.00^AY^
14	54.10 ± 1.56^AB^	56.20 ± 1.98^bc^	55.80 ± 1.41^AB^	56.70 ± 0.42^B^	53.80 ± 0.28^B^	54.80 ± 0.57^B^	56.80 ± 0.00^B^	55.60 ± 0.57^B^
21	57.20 ± 0.00^CL^	57.20 ± 0.00^CL^	55.80 ± 0.28^ABZT^	56.00 ± 0.00^ABTK^	55.00 ± 0.00^CYZ^	53.60 ± 0.00^AX^	54.60 ± 0.85^BY^	56.80 ± 0.57^CKL^
28	56.00 ± 0.00B^CT^	54.80 ± 0.00^ABZ^	56.80 ± 0.00B^L^	58.00 ± 0.00^CM^	56.40 ± 0.00^DK^	54.80 ± 0.00^BZ^	54.40 ± 0.00^BY^	52.00 ± 0.00^AX^

*Note:* ^A,B,C,D,E^: Different letters in the same column indicate differences at *p* < 0.05 level. ^X,Y,Z,T,K,L,M,N^: Different letters on the same line indicate difference at *p* < 0.05 level.

Abbreviations: CC, *Candida colliculosa* LAF 7; DH, *Debaryomyces hansenii* LAF 3; GC, *Geotrichum candidum* GEO; KL, *Kluyveromyces lactis* PYCC 4356; KM, *Kluyveromyces marxianus* LAF 4; SB, *Saccharomyces boulardii* CNCM I‐745; SC, *Saccharomyces cerevisiae*; YL, *Yarrowia lipolytica* NCAIM Y00591.

When the viscosity values obtained in the samples are examined, it is seen that the values obtained between the samples both during storage and on the storage days are statistically significant (*p* < 0.05). During the 28‐day storage period, the value of viscosity in all samples initially increased and tended to decrease at the end of storage. Maximum viscosity values were reported on the 14th storage day. While the viscosity values measured for all kefir samples are reported as 478.00–3218.50 cP, it can be seen that the highest and lowest values belong to the DH sample.

No serum separation (syneresis) was observed in any of the kefir samples during the 28‐day storage period. Although kefir is a dairy product with high water content, the bacteria in the kefir microbiota and the yeast species used were in harmony, and the pH did not exceed a certain level, so no serum separation problem occurred. Although yeasts are important flavor parameters in kefir production, they also affect quality parameters such as viscosity and serum separation. Although it has been reported that the most common yeasts in kefir beverages produced using grain are *Saccharomyces kefiri*, *K. lactis*, and *Torulopsis* spp. (Zafar and Owais 2006; Buran 2020), yeast species that do not metabolize lactose or metabolize it poorly are not preferred in industrial kefirs in order to preserve shelf life and prevent excess gas formation. For this purpose, it is important to investigate the effect of yeast combinations with bacteria as secondary cultures on the final product quality. In a study evaluating the quality and aroma properties of kefir produced using grain and lyophilized culture, viscosity values in kefir samples varied between 124.58 and 127.98 cP during the 21‐day storage period. It was stated that the viscosity value was higher in kefir produced with grain (Dag et al. 2021). The culture combination used in kefir production has a very important effect on the quality parameters of the final product. Simova et al. (2002) evaluated the quality parameters of samples produced from kefir grains, direct kefir and (83%–90%) lactic acid bacteria found in kefir. While it was emphasized in the study that the viscosity value of kefir produced with grains was higher, viscosity values were reported in the range of 1043–1075 cst.

The viscosity and serum separation values in the production of fermented milk beverages are related to the amount of dry matter and protein content of the product. Exopolysaccharides (EPS) produced by microorganisms used as cultures in kefir production bind the water in the structure and provide stability to the final product. In this way, while a decrease in the amount of separation is expected, an increase in viscosity values is expected (Duboc and Mollet 2001; Tamime et al. 2007). Although hardness and viscosity are generally considered inversely proportional in dairy systems, both parameters increased in some kefir samples toward the end of the storage period. In this study, hardness was evaluated as a comparative parameter among the samples rather than a standalone quality criterion. Overall, increases in viscosity were found to be associated with corresponding increases in hardness values.

In our study, serum separation was not detected in kefir samples produced with the combination of yeast and lactic acid bacteria during 28 days of storage. When WHC values were compared, syneresis was not seen in any sample. This can be attributed to the fact that the pH values of the kefirs did not fall below a certain level and that the lactic acid bacteria and yeasts in the microbiota were in harmony. Serum separation is considered a critical quality parameter in fermented dairy products due to its influence on consumer acceptance. In industrial applications, this issue is often addressed through the incorporation of additives—such as stabilizers or hydrocolloids—which help improve product stability and minimize phase separation. In many studies in the literature, samples with serum separation in sensory evaluations of fermented dairy products are interpreted as being less liked by the panelists (Bodyfelt et al. 2009; Bekis 2019; Saygili et al. 2021). Serum separation was not detected in kefir samples, showing that the acceptability of the products by the consumer will be higher than the samples with serum separation. WPC in fermented dairy products can be defined as protein–water interaction. Although WPC is closely related to viscosity, it can be affected by many parameters (pH, heat treatment norms, fermentation conditions, etc.) that affect the functional properties of milk proteins (Li et al. 2016; Seo and Oh 2023). In the present study, WHC values were determined in the range of 51.30%–62.80%. While there were fluctuations in the water‐retention capacity values of kefir samples throughout storage, the change was determined to be statistically significant (*p* < 0.05). Unal et al. (2020) in the study titled determination of some quality parameters of commercial kefir, the water‐retention capacity values obtained were expressed as 29.45%–38.75%. The data obtained in the study were determined to be above this value. This is interpreted as the fact that only lactic acid bacteria are used in the production of commercial kefir cultures and therefore their ability to produce EPS is lower than kefir containing yeast. In addition to the effect of the starter culture used in production, WHC can be increased with practices such as heat treatment norms and homogenization applied to milk, and thus, it is reported that an increase in viscosity values is recorded (Atamer and Sezgin 1987).

### 3.5 CO_2_ Contents and Acidity Index

Since the development and metabolic activities of yeasts cannot be limited like bacteria during the storage of kefir, an increase in the amount of CO_2_ is observed due to yeast activity during storage. Considering that excessive CO_2_ production will cause some negative taste and aroma defects in kefir, it is thought that the increase in CO_2_ should be controlled. The amount of CO_2_ produced and the acidity index value in kefir produced using different yeast types are given in Table 5. When the statistical evaluation of the CO_2_ amount values produced by kefirs was made, the change in each product during storage was found to be significant (*p* < 0.05), and the change between the products on each storage day was found to be significant (*p* < 0.05).

**TABLE 5:** CO_2_ contents and acidity index values determined in kefir drinks.

Storage days	Kefir samples							
	DH	KM	CC	YL	SB	SC	KL	GC
	CO_2_ (mg/100 mL)							
1	3.52 ± 0.93^AY^	14.01 ± 0.62^ATK^	9.24 ± 1.56^AZ^	15.45 ± 0.14^AK^	0.98 ± 0.78^AX^	12.49 ± 0.62^AT^	2.86 ± 0.62^AXY^	2.53 ± 0.16^AXY^
7	6.49 ± 1.09^AY^	15.06 ± 0.0^BT^	11.77 ± 0.47^AZ^	15.32 ± 0.20^AT^	1.43 ± 0.78^AX^	14.67 ± 0.28^BT^	6.71 ± 1.09^AY^	5.17 ± 0.16^ABY^
14	16.95 ± 1.54^BZT^	18.64 ± 0.01^CT^	11.99 ± 1.71^AY^	15.04 ± 0.01^AZ^	7.59 ± 0.78^AX^	15.26 ± 0.30^BZ^	8.25 ± 0.16^AX^	9.54 ± 0.20^BX^
21	38.31 ± 0.35^CZ^	17.52 ± 0.06^DX^	17.38 ± 1.56^BX^	15.59 ± 0.32^AX^	31.01 ± 0.64^BY^	15.43 ± 0.37^BX^	34.32 ± 5.29^BYZ^	36.96 ± 0.62^CZ^
28	108.35 ± 4.51^DZ^	118.94 ± 0.42^EZ^	80.84 ± 2.12^CY^	66.16 ± 0.42^BX^	136.12 ± 7.55^CT^	151.40 ± 1.07^CK^	140.47 ± 9.18^CTK^	83.05 ± 5.76^CY^
	Acidity index (mg KOH/100 g)							
1	3.54 ± 0.06^XY^	3.12 ± 0.26^AXY^	3.47 ± 0.04^AXY^	3.71 ± 0.18^Y^	3.19 ± 0.24^AXY^	4.88 ± 0.12^DZ^	3.50 ± 0.52^AXY^	2.99 ± 0.12^AX^
7	3.44 ± 0.20^X^	5.19 ± 0.12^CZ^	4.39 ± 0.14^CY^	3.64 ± 0.20^X^	4.23 ± 0.16^CY^	3.43 ± 0.06^BX^	5.10 ± 0.36^CZ^	3.47 ± 0.16^BX^
14	3.57 ± 0.02^XYZ^	3.98 ± 0.36^BZ^	3.37 ± 0.10^AXY^	3.71 ± 0.22^YZ^	3.78 ± 0.12^BYZ^	3.21 ± 0.01^AX^	4.53 ± 0.26^BCK^	3.54 ± 0.14^BXYZ^
21	3.29 ± 0.19^X^	4.02 ± 0.10^BZT^	3.79 ± 0.02^BYZ^	3.26 ± 0.02^X^	3.58 ± 0.12^ABY^	3.67 ± 0.04^CY^	4.09 ± 0.20^ABT^	3.55 ± 0.12^BY^
28	3.59 ± 0.08^XY^	4.68 ± 0.16^CK^	3.50 ± 0.04^AX^	3.91 ± 0.02^Z^	3.47 ± 0.20^ABX^	3.75 ± 0.08^CYZ^	4.15 ± 0.04^ABT^	3.54 ± 0.16^BXY^

*Note:* ^A,B,C,D,E^: Different letters in the same column indicate differences at *p* < 0.05 level. ^X,Y,Z,T,K,L,M,N^: Different letters on the same line indicate difference at *p* < 0.05 level.

Abbreviations: CC, *Candida colliculosa* LAF 7; DH, *Debaryomyces hansenii* LAF 3; GC, *Geotrichum candidum* GEO; KL, *Kluyveromyces lactis* PYCC 4356; KM, *Kluyveromyces marxianus* LAF 4; SB, *Saccharomyces boulardii* CNCM I‐745; SC, *Saccharomyces cerevisiae*; YL, *Yarrowia lipolytica* NCAIM Y00591.

CO_2_ and alcohol, which are formed as a result of the metabolite activities of yeasts, are the most important components of kefir, and the amount of acidity, CO_2_, and alcohol in kefir varies depending on storage conditions. The microbiota of kefir ferments milk and creates lactic acid, CO_2_, small amounts of alcohol, acetaldehyde, acetone, and diacetyl, contributing to the formation of kefir's unique aroma and consistency. A good kefir should contain 50 g/100 mL CO_2_ (by volume) (Toba et al. 1987; Guzel‐Seydim et al. 2000; Ozden 2008; Karatepe et al. 2012). Depending on the type of yeast used, increases in the amount of CO_2_ were observed in each kefir in the following days of storage. The highest increase occurred only on the last day of storage for all products. When we examined each storage day in terms of the changes occurring between the products, it was seen that on the first day of storage, kefir samples containing *K. marxianus*, *C. colliculosa*, *Y. lipolytica*, and *S. cerevisiae* created higher levels of CO_2_ than kefir samples containing other yeast species. The lowest CO_2_ formation is seen in the sample containing *S. boulardii*. CO_2_ formation progressed slowly in all samples on the 7th and 14th storage days, but on the 21st day, the increase in samples containing *D. hansenii* , *S. boulardii*, *K. lactis*, and *Geo. candidum* was observed to be higher than in the others. On the last day of storage, it increased to 108.35–151.40 mg/100 mL in samples containing *S. cerevisiae* , *K. lactis*, *S. boulardii*, *K. marxianus*, and *D. hansenii* , respectively. Samples containing *C. colliculosa*, *Y. lipolytica*, and *G. candidum* increased to 66.16–83.05 mg/100 mL, and it is thought that these samples, except for *G. candidum* and including *K. marxianus*, will not cause any problems in packaging until the 1st, 7th, 14th and 21st days of storage. For all other samples, it is thought that the same problem will not occur until the 1st, 7th and 14th days.

Tomar (2015), who obtained similar results to our study, found that the CO_2_ amount of kefir produced with milk containing different amounts of fat was 44.66–83.48 mg/100 mL at the beginning of storage, and the CO_2_ amounts of the samples increased throughout storage, reaching up to 116.03 mg/100 mL. Yildiz (2009) stated that the amount of CO_2_ in kefirs produced with milk with different fat contents varied between 57.4 and 207.7 mg/100 mL during storage and that the amount of CO_2_ increased as the storage time increased. Alpkent and Kucukcetin (2000) found that the CO_2_ amount of the kefir they produced increased from 10.52 mg/100 mL to 188.70 mg/100 mL during the 21‐day storage period. Although CO_2_ content is used to classify kefirs as weak, medium, and strong, it also has a positive effect on the taste and aroma of the product (Gursel et al. 1990). Rossi and Gobetti (1991) determined that the average CO_2_ amount of kefir produced with starter culture containing *S. unisponus*, *S. italicus* , *L. kefir*, *L. kefiranofaciens* , *L. lactis* , and *E. durans* was 0.44 g/L (44% mg/100 mL). Simova et al. (2002) found that the CO_2_ amount of kefir production from kefir grains and kefir produced from kefir grains in which kefir was used as inoculum was 85% and 15%, respectively. Yilmaz et al. (2006) stated that the amount of CO_2_ in plain kefir produced using kefir grains increased by 132.40%–286% during 10 days of storage. Konar and Sahan (1989) stated that the amount of CO_2_ in kefir produced using different types of milk varies between 93% and 227%. Clementi et al. (1989) reported that yeasts and some LAB were responsible for CO_2_ production in kefir.

The acidity value, which expresses the amount of free fatty acids (FFAs) in fermented dairy products, is effective in the formation of the flavor and aroma of the product. These occur as a result of culture‐derived lipolytic activity from milk fat, as well as lactose transformation and oxidative deamination, transamination, and decarboxylation of amino acids (Akdan et al. 2020). As a result of the statistical evaluation made on kefirs using different yeast species, the change in acidity index values of kefirs during storage was found to be significant in all samples (*p* < 0.05), except for DH and YL samples (*p* > 0.05). The change between samples on each storage day was found to be significant in all samples (*p* < 0.05). While the acidity index value did not change in some products during storage, an increase was observed in some products (KM, CC, SB, and KL) on the 7th storage day. In the last days of storage, variations in the form of increases and decreases are observed. The variable course of acidity index values, especially during storage processes, is due to the conversion of low‐molecular free fatty acids into components such as esters and alcohols (Akdan et al. 2020). Differences in the yeast types used also cause changes in the amount of free fatty acids in kefir depending on lipolytic activity. It is seen that kefirs containing *K. marxianus* and *K. lactis* have higher acidity index values. The acidity index value of all kefirs varies between 3.12 and 5.19 mg KOH/100 g during storage. While the highest value is seen in kefir containing *S. cerevisiae* on the 1st day of storage, it is seen in kefir containing *K. marxianus* and *K. lactis* on the 7th, 14th, 21st and 28th day (3.98–5.19 mg KOH/100 g). In other samples, it varied between 3.29 and 3.91 mg KOH/100 g throughout the entire storage period. Akdan et al. (2020) produced kefir with buffalo milk and other milk mixtures in their study and found that the acidity index value in kefir varied between 1.60 and 2.15 mg KOH/100 g. Oliveira et al. (2018) inoculated a water emulsion containing coconut oil with *C. kefyr* and it was determined that the acidity index (FFAs) varied in a wide range of 0.95–12.56 KOH/g. Evaluation of the results indicates that acidity index values—reflecting the concentration of free fatty acids—vary depending on both the species and population density of the yeasts present in the product.

### 3.6 Microbiological Properties

*Lactococcus* spp./ *S. thermophilus* counts in all kefirs decreased during storage (*p* < 0.05). Although the differences between the samples are statistically significant (*p* < 0.05), the highest viability is in the CC sample. When *L. cremoris* viability was examined, a decreasing trend was observed in all samples, while the highest end‐of‐storage viability was determined in the GC and YL samples. The most pronounced decrease in a given sample was observed in the DH sample. Additionally, yeast counts increased across all samples during storage, with the DH sample showing the highest increase relative to the initial values. In the SB and SC samples, changes in the number of yeasts remained limited. All samples showed growth above the 4 log level at the beginning and end of storage. UHT milk was used in kefir production, and aseptic work was carried out to prevent yeast contamination during the production and packaging stages. It was observed that the differences in yeast numbers were statistically significant (*p* < 0.05) and that this varied depending on whether the yeasts in the culture could ferment lactose (Table 6). Variations in yeast populations are closely associated with changes in CO_2_ production and the carbohydrate composition of kefir. In addition, keeping the yeast numbers at certain levels is important both for product quality and for preventing any negative effects that may occur during storage (Riesute et al. 2021). In general, it is important that the yeast contents in all samples remain at or below the highest level of 7 log CFU/mL at the end of storage. Yeast growth above these levels can lead to problems such as quality problems and packaging deformation in kefirs (Alvarez‐Martin et al. 2008; Buchl and Seiler 2011). There was no packaging damage or problems such as swelling or foaming in any samples. No mold growth was seen in any sample; only the GC sample showed growth of distinct looking yeast colonies (mold‐like).

**TABLE 6:** Microbiological properties of kefir drinks (log CFU/mL).

Storage days	Kefir samples							
	DH	KM	CC	YL	SB	SC	KL	GC
	*Lactococcus* spp./ *Streptococcus thermophilus*							
1	7.92 ± 0.13^YZ^	7.87 ± 0.04^BY^	8.17 ± 0.05^BZT^	7.95 ± 0.19^BK^	8.10 ± 0.11^CYZT^	7.77 ± 0.33^BX^	7.97 ± 0.14^BCYZ^	8.26 ± 0.03^DTK^
7	7.87 ± 0.17^X^	7.81 ± 0.40^BX^	7.43 ± 0.28^AX^	7.55 ± 0.07^BY^	8.58 ± 0.25^DY^	7.83 ± 0.04^BX^	7.49 ± 0.16^AX^	7.85 ± 0.02^CX^
14	7.66 ± 0.35^XY^	7.92 ± 0.00^BYZ^	7.51 ± 0.00^AX^	7.48 ± 0.19^AX^	7.47 ± 0.12^BX^	7.74 ± 0.03C^XY^	8.15 ± 0.08^CZ^	7.62 ± 0.04^BXY^
21	7.74 ± 0.12^T^	6.83 ± 0.18^AX^	7.85 ± 0.23^BT^	7.29 ± 0.02^AZ^	7.06 ± 0.08^AY^	7.22 ± 0.06^AYZ^	7.86 ± 0.06^BT^	7.12 ± 0.12^AYZ^
28	7.79 ± 0.02^K^	6.91 ± 0.02^AX^	7.89 ± 0.03^BL^	7.41 ± 0.04^AZ^	7.40 ± 0.02^ABZ^	7.11 ± 0.05^AY^	7.76 ± 0.03^BK^	7.51 ± 0.04^BT^
	*Leuconostoc mesenteroides* subsp. *cremoris*							
1	7.11 ± 0.05^B^	7.10 ± 0.46^C^	7.43 ± 0.28^C^	7.31 ± 0.27^C^	7.28 ± 0.39^C^	7.27 ± 0.10^A^	7.60 ± 0.04^A^	7.45 ± 0.10^C^
7	7.20 ± 0.04^BXY^	7.04 ± 0.00^CX^	7.19 ± 0.02^CXY^	7.05 ± 0.07^CYZ^	7.76 ± 0.15^CZ^	6.90 ± 0.14^AX^	6.93 ± 0.21^BX^	7.20 ± 0.28^BCXY^
14	6.11 ± 0.10^AXY^	6.84 ± 0.01^BCT^	6.61 ± 0.06^AX^	6.65 ± 0.20^AX^	6.37 ± 0.31^BYZ^	6.44 ± 0.14^AYZ^	6.56 ± 0.03^AZ^	6.83 ± 0.03^BT^
21	6.08 ± 0.18^AY^	6.44 ± 0.08^ABZ^	5.87 ± 0.04^BT^	6.04 ± 0.00^BT^	5.38 ± 0.11^AX^	6.35 ± 0.08^BT^	6.55 ± 0.01^CZT^	6.09 ± 0.12^AY^
28	5.94 ± 0.01^AX^	6.29 ± 0.05^AY^	6.69 ± 0.05^BK^	6.77 ± 0.03^BL^	6.60 ± 0.01^BT^	6.58 ± 0.03^BT^	6.46 ± 0.02^AZ^	6.80 ± 0.04^BM^
	Yeast—molds							
1	4.29 ± 0.02^AX^	5.39 ± 0.04^AK^	4.10 ± 0.03^BT^	4.05 ± 0.07^AX^	4.45 ± 0.08^Z^	4.41 ± 0.01^AX^	5.33 ± 0.11^ABK^	4.48 ± 0.67^BY^
7	4.61 ± 0.15^BY^	5.55 ± 0.04^AK^	4.91 ± 0.21^ABT^	4.19 ± 0.07^AX^	4.50 ± 0.06^Z^	4.51 ± 0.01^BY^	5.57 ± 0.25^AK^	4.63 ± 0.21^AY^
14	5.20 ± 0.14^CT^	5.63 ± 0.54^AK^	4.93 ± 0.06^AZ^	5.18 ± 0.00^BY^	4.52 ± 0.03^Z^	4.54 ± 0.01^CX^	5.80 ± 0.00^BK^	4.94 ± 0.34^CY^
21	6.94 ± 0.10^DL^	6.31 ± 0.19^BK^	6.02 ± 0.02^CT^	6.04 ± 0.20^CTK^	4.56 ± 0.00^Z^	4.61 ± 0.01^DX^	6.87 ± 0.04^CL^	5.13 ± 0.06^CY^
28	7.00 ± 0.03^DM^	6.54 ± 0.07^BK^	7.11 ± 0.03^DN^	6.29 ± 0.05^CT^	4.70 ± 0.31^Z^	4.68 ± 0.03^EX^	6.89 ± 0.01^CL^	5.18 ± 0.01^CY^

*Note:* ^A,B,C,D,E^: Different letters in the same column indicate differences at *p* < 0.05 level. ^X,Y,Z,T,K,L,M,N^: Different letters on the same line indicate difference at *p* < 0.05 level. *Lactococcus* spp.: *Lactococcus lactis* subsp. *Lactis*; *Lactococcus lactis* subsp. *cremoris*; *Lactococcus lactis* subsp. *lactis* Biovar *diacetylactis*.

Abbreviations: CC, *Candida colliculosa* LAF 7; DH, *Debaryomyces hansenii* LAF 3; GC, *Geotrichum candidum* GEO; KL, *Kluyveromyces lactis* PYCC 4356; KM, *Kluyveromyces marxianus* LAF 4; SB, *Saccharomyces boulardii* CNCM I‐745; SC, *Saccharomyces cerevisiae*; YL, *Yarrowia lipolytica* NCAIM Y00591.

In a study on the use of LAB and yeasts in kefir, LAB was added to milk first and then yeast, and as a result of this application, differences were caused in the numbers of *L. lactis* , *S. thermophilus* , and *Lactobacillus* spp. in kefir (Beshkova et al. 2003) Guzel‐Seydim et al. (2005) produced kefir with starter culture. They determined the yeast counts in these kefirs as 4.77 log CFU/mL on the 1st day, 4.81 log CFU/mL on the 7th day, and 5.00 log CFU/mL on the 21st day. They determined that the number of yeasts increased during storage. In another study, they found that in kefir, whose yeast count was initially 10^3^ CFU/mL, the yeast count increased to 1.5 × 10^6^ CFU/mL after 2 days of storage (Rea et al. 1996). Yildiz‐Akgul et al. (2018) produced kefir from milk containing 3% fat and stored it for 23 days. Storage yeast count 1st day 7.11 log CFU/mL, 5th day 8.08 log CFU/mL, 10th day 8.27 log CFU/mL, 16th day 8.45 log CFU/mL, and 23rd day, it was determined to be 8.16 log CFU/mL. High yeast results increase depending on the yeast and content density used in kefir production. This may or may not cause problems depending on the gas‐forming potential of the yeast. Beshkova et al. (2003, in their study, found the number of yeasts in kefir produced with pure culture to be 6 log CFU/mL during the 24‐h ripening phase of kefir at 4°C after fermentation. Simova et al. (2002) determined that the yeast counts they found in the kefir they produced from kefir grains and filtrate were 5.30 log CFU/mL and 4 log CFU/mL, respectively. Alpkent and Kucukcetin (2000) examined the changes in kefir made with grains stored at different temperatures. In this study, they determined the amount of yeast in kefir samples stored at 5°C as 5.34 log CFU/mL, 5.85 log CFU/mL, and 5.68 log CFU/mL on days 1, 9, and 15, respectively. It was stated that the amounts of microorganisms in kefir grain, kefir culture, and kefir drink were different, and the yeast numbers were variable. However, there have been no reports of yeast survival being a problem at these high levels (Simova et al. 2002; Gao and Li 2016; Ding et al. 2022; Xu et al. 2023).

### 3.7 Sensory Features

As with all fermented milk products, taste and aroma are important parameters for kefir quality. The average taste scores determined in kefir samples were highest in CC samples, followed by SB and SC samples (Table 7). In general, the KM sample also had an acceptable score in terms of taste. When compared in terms of physicochemical properties, it is thought that the CO_2_ acidity indexes of the KM sample are at a certain level and are effective in terms of sensory appeal. In the CC sample, a stable acidity increase caused it to have the highest scores in terms of taste. Considering the acidity and other properties of kefir samples, the acid formation and alcohol fermentation in kefir provide kefir with its unique taste and aroma (Marshall and Cole 1985; Marquina et al. 2002; Beshkova et al. 1998; Otles and Çagindi 2003). Kefir is a refreshing and enjoyable drink with a slightly sour and yeasty taste. Therefore, the ratios of lactic acid bacteria and yeasts play the biggest role in the formation of the desired taste and aroma. This situation is supported by the microbiological properties of kefirs and therefore their bacterial–yeast development. Uslu (2010) on fruity, plain, and light kefirs sold in the market, the average consistency scores were between 6.66 and 7.69. Following the decrease in taste–aroma scores during storage, Irigoyen et al. (2005) reported that the acceptability level of two types of kefir samples during storage was highest in the first days of storage and decreased with increasing storage time. Gungor (2007) reported that a decrease in the color, taste, and appearance characteristics of kefir was detected in all samples at the end of storage, depending on the storage time. This decrease during storage may be due to the increase in acidity of kefir samples and the metabolites produced by the increasing yeast numbers.

**TABLE 7:** Sensory properties of kefir drinks.

Storage days	Kefir samples							
	DH	KM	CC	YL	SB	SC	KL	GC
	Taste							
1	7.80 ± 1.64	7.60 ± 0.89^B^	8.20 ± 0.84	7.60 ± 0.89	8.20 ± 0.84	8.20 ± 0.84^C^	7.80 ± 1.10^B^	7.80 ± 0.84^B^
7	7.00 ± 1.22	6.20 ± 1.48^AB^	8.00 ± 0.00	7.60 ± 1.14	7.60 ± 1.14	8.00 ± 1.00^bc^	7.60 ± 0.89^B^	7.60 ± 1.14^B^
14	7.00 ± 0.71^XYZ^	5.60 ± 1.14^AX^	6.80 ± 0.84^XYZ^	7.60 ± 0.89^Z^	7.20 ± 1.30^YZ^	6.00 ± 1.22^AXY^	6.80 ± 1.30B^XYZ^	7.60 ± 0.55^BZ^
21	6.60 ± 1.14	6.20 ± 0.84^AB^	7.40 ± 1.14	7.00 ± 1.00	7.00 ± 1.00	7.00 ± 0.00^ABC^	6.60 ± 0.55^B^	7.00 ± 0.00^AB^
28	6.80 ± 1.30^Y^	7.20 ± 0.84^BY^	7.40 ± 0.55^Y^	6.40 ± 1.67^Y^	6.60 ± 1.52^Y^	6.80 ± 0.84^ABY^	4.40 ± 0.55^AX^	5.80 ± 0.00^AXY^
	Structure—consistency							
1	8.60 ± 0.55	8.60 ± 0.55^B^	8.00 ± 1.73	8.60 ± 0.55^B^	8.80 ± 0.45^B^	8.40 ± 0.89^C^	8.40 ± 1.34^B^	8.20 ± 0.84^C^
7	7.20 ± 1.92	6.60 ± 1.67^A^	7.60 ± 1.52	8.00 ± 0.00^AB^	7.80 ± 1.10^AB^	7.40 ± 0.89^ABC^	7.80 ± 0.45^AB^	7.80 ± 0.84^bc^
14	7.40 ± 0.55	7.40 ± 0.89^AB^	7.80 ± 1.10	7.60 ± 0.89^AB^	7.80 ± 0.45^AB^	7.80 ± 0.45^bc^	7.60 ± 0.55^AB^	7.60 ± 0.55^bc^
21	7.40 ± 0.89	6.80 ± 0.45^A^	7.20 ± 0.84	7.00 ± 1.00^A^	6.60 ± 0.55^A^	6.40 ± 0.55^A^	6.80 ± 1.10^A^	6.40 ± 0.55^A^
28	6.80 ± 1.10	7.20 ± 0.84^A^	7.60 ± 0.89	7.40 ± 0.89^A^	7.20 ± 1.48^A^	7.20 ± 0.84^AB^	6.60 ± 0.55^A^	6.80 ± 1.10^AB^
	Smell—aroma							
1	6.80 ± 2.17	7.60 ± 0.89	8.40 ± 0.55	8.00 ± 0.71^B^	8.60 ± 0.55^C^	8.20 ± 0.45^B^	8.60 ± 0.55^D^	8.00 ± 0.71
7	7.40 ± 0.55	7.00 ± 0.71	7.80 ± 0.45	8.20 ± 0.45^B^	8.00 ± 0.00^C^	8.00 ± 0.71^B^	7.60 ± 0.89^C^	8.00 ± 0.71
14	7.40 ± 0.55^Y^	6.40 ± 0.89^X^	7.80 ± 0.84^Y^	8.00 ± 0.00^BY^	7.80 ± 0.45^BCY^	7.80 ± 0.45^BY^	7.40 ± 0.89^BCY^	7.60 ± 0.89^Y^
21	6.60 ± 0.55	6.40 ± 0.55	7.40 ± 0.55	6.80 ± 1.30^AB^	6.60 ± 0.55^A^	6.60 ± 0.55^A^	6.40 ± 0.55^B^	7.00 ± 0.00
28	6.80 ± 1.10^Y^	7.20 ± 1.10^Y^	7.40 ± 0.55^Y^	6.20 ± 1.92^AY^	7.00 ± 1.22^ABY^	7.00 ± 0.71^AY^	4.00 ± 1.22^AX^	6.80 ± 1.30^Y^
	General acceptability							
1	7.80 ± 1.64	8.00 ± 1.22	8.40 ± 0.55	8.20 ± 0.84	8.40 ± 0.55^C^	8.60 ± 0.55^C^	8.40 ± 1.34^C^	7.80 ± 0.84^B^
7	7.40 ± 0.89	6.80 ± 1.30	8.00 ± 0.00	8.00 ± 0.71	7.60 ± 0.55^ABC^	7.80 ± 0.84^BC^	7.60 ± 0.89^bc^	7.80 ± 0.84^B^
14	7.20 ± 0.45^Y^	5.80 ± 1.30^X^	8.00 ± 1.00^Y^	8.00 ± 0.71^Y^	7.80 ± 0.84^bcY^	7.20 ± 0.84^ABY^	7.00 ± 1.22^BY^	7.80 ± 0.45^BY^
21	7.40 ± 0.55	7.20 ± 0.84	7.20 ± 0.84	7.40 ± 0.55	6.60 ± 0.55^A^	7.00 ± 0.00^AB^	7.00 ± 0.00^B^	7.00 ± 0.00^B^
28	6.60 ± 0.89^YZ^	7.20 ± 0.84^Z^	7.40 ± 0.55^Z^	6.40 ± 1.67^YZ^	6.80 ± 1.30^ABYZ^	6.80 ± 0.84^AYZ^	4.20 ± 0.45^AX^	5.40 ± 1.67^AXY^

*Note:* ^A,B,C,D,E^: Different letters in the same column indicate differences at *p* < 0.05 level. ^X,Y,Z,T,K,L,M,N^: Different letters on the same line indicate difference at *p* < 0.05 level.

Abbreviations: CC, *Candida colliculosa* LAF 7; DH, *Debaryomyces hansenii* LAF 3; GC, *Geotrichum candidum* GEO; KL, *Kluyveromyces lactis* PYCC 4356; KM, *Kluyveromyces marxianus* LAF 4; SB, *Saccharomyces boulardii* CNCM I‐745; SC, *Saccharomyces cerevisiae*; YL, *Yarrowia lipolytica* NCAIM Y00591.

While there was no significant difference between kefir samples in terms of structure and consistency, the KM sample received a higher score. The CC sample received a high average score in terms of odor criterion, and the scores of the KL sample decreased during storage and reached the lowest level at the end of storage. When the viscosity and hardness values of kefirs and their sensory scores are taken into consideration, it supports high scores in the CC and KM samples. The characteristics of the microbiota in fermented milk products, dry matter content, and other physicochemical properties affect the structural properties. The differences between the samples are natural due to the use of different yeasts. Considering that the raw material and kefir culture used are standard, the yeast species and strains used affect this difference (*p* < 0.05). When compared with the hardness and viscosity measurements performed instrumentally, the structure‐consistency scores also showed parallelism. The CC, KM, and SB samples with higher hardness and viscosity values managed to get higher sensory scores. The consistency and structural properties of kefir are affected by starter culture type and activities, fermentation conditions (temperature, duration, and final pH), and storage conditions (Lucey 2004). In addition, in similar kefir studies made from different types of milk and their mixtures, it was emphasized that the contribution of the mechanical effect to the consistency and sticky properties of kefir during the preservation process is important (Cais‐Sokolińska et al. 2016). Kefir should have a structurally homogeneous, fluid, and shiny appearance and should not have a lumpy structure.

The highest scores in terms of smell‐aroma scores were in CC and KM samples. The specific smell and aroma of kefir are provided by the harmony of LAB and yeasts and the activity of the yeasts. In fermented milk containing yeast such as kefir, a high yeast taste and aroma are not desired, and low aroma is not desired either. In addition, kefir acidity and pH also affect the taste and aroma. Although creamy aroma, viscous structure, and starter cultures are effective in determining the sensory character of kefir beverages, it is reported in the literature that the main factors are milk type and storage period (Wszolek et al. 2001).

In the last criterion where the general acceptability was made, the panelists made an evaluation close to the taste–aroma criteria and liked the CC coded sample the most. LAB is more effective than yeast in the formation of taste and aroma substances during fermentation. In kefir fermentation, yeasts play an important role in creating an acidic, refreshing, and slightly yeasty taste. In good‐tasting kefir, acidity and a refreshing and slightly yeast‐like kefir taste occur during fermentation, especially under the influence of CO_2_ created by the yeast (Irigoyen et al. 2005). The sharp acid taste and yeasty taste of kefir are due to the CO_2_ produced by the yeast. It is the yeast flora that gives kefir its typical flavor.

The sensory properties and quality are directly dependent on the consumer and play an important role in the marketing of products. As in many fermented foods, LAB and yeasts have the biggest impact on the aroma and smell formation in kefir. Microorganisms and the metabolites formed by microorganisms play an important role in the sensory profile of kefir during the fermentation period (Wong et al. 2018; Guangsen et al. 2021). Metabolites produced by yeasts, which provide the necessary environment for the development of kefir bacteria, also contribute to the taste and flavor of kefir in the mouth (Garrote et al. 2010).

## 4 Conclusion and Recommendations

In recent years, importance has been given to the development of starter cultures for the production of kefir beverages similar to traditional kefir produced using grains. However, due to the characteristics of the yeast used in production, not every yeast can be used or is used in a limited way. In this study, where different yeast species were used in the production of kefir drink, it was observed that the yeasts in the starter culture composition affected the product properties due to interaction with different yeasts and cultures. A relatively higher amount of CO_2_ was observed in kefirs containing *S. cerevisiae* and *S. boulardii*, but these amounts did not affect the packaging and appearance characteristics of the products during storage. It was observed that especially kefirs containing *K. marxianus* and *K. lactis* had higher acidity index values. In general, it is important in this respect that the yeast contents in all samples remain at or below the highest level of 7 Log CFU/mL at the end of storage. In addition, the best sensory properties occurred in kefirs produced using *Candida colliculosa*. It is recommended to conduct studies on the dual use of these yeast species in future studies.

## Author Contributions

**Oktay Yerlikaya:** conceptualization (equal), data curation (equal), formal analysis (equal), funding acquisition (equal), investigation (equal), methodology (equal). **Asli Akpinar:** methodology (equal), writing – original draft (equal). **Derya Saygili:** validation (equal), writing – original draft (equal).

## Conflicts of Interest

The authors declare no conflicts of interest.

## Data Availability

No data were used for the research described in the article.
